# The mTOR Pathway in Pluripotent Stem Cells: Lessons for Understanding Cancer Cell Dormancy

**DOI:** 10.3390/membranes11110858

**Published:** 2021-11-07

**Authors:** Bashar A. Alhasan, Sergei A. Gordeev, Aleksandra R. Knyazeva, Kseniia V. Aleksandrova, Boris A. Margulis, Irina V. Guzhova, Irina I. Suvorova

**Affiliations:** Institute of Cytology, Russian Academy of Sciences, 194064 St. Petersburg, Russia; alhassan.bashar.1994@gmail.com (B.A.A.); s.a.gordeev@hotmail.com (S.A.G.); aleksandra.knyaz@mail.ru (A.R.K.); aleksandrova_k_v@mail.ru (K.V.A.); margulis@incras.ru (B.A.M.); irina.guzh@gmail.com (I.V.G.)

**Keywords:** embryonic stem cells, embryonic diapause, pluripotent stem cells, pluripotency, cancer cell dormancy, autophagy, mTOR

## Abstract

Currently, the success of targeted anticancer therapies largely depends on the correct understanding of the dormant state of cancer cells, since it is increasingly regarded to fuel tumor recurrence. The concept of cancer cell dormancy is often considered as an adaptive response of cancer cells to stress, and, therefore, is limited. It is possible that the cancer dormant state is not a privilege of cancer cells but the same reproductive survival strategy as diapause used by embryonic stem cells (ESCs). Recent advances reveal that high autophagy and mTOR pathway reduction are key mechanisms contributing to dormancy and diapause. ESCs, sharing their main features with cancer stem cells, have a delicate balance between the mTOR pathway and autophagy activity permissive for diapause induction. In this review, we discuss the functioning of the mTOR signaling and autophagy in ESCs in detail that allows us to deepen our understanding of the biology of cancer cell dormancy.

## 1. Introduction

Pluripotent cells can indefinitely divide in vitro under certain conditions retaining their undifferentiated state, although pluripotency in vivo is transient and becomes limited shortly after implantation. An exception from this rule is embryonic diapause—a period of embryonic suspension at the blastocyst stage caused by unfavorable conditions. Diapause is a physiological reproductive strategy that is widespread in various animals (for example, in mice, but not in humans). Therefore, mouse ESCs (mESCs) isolated from blastocysts during diapause, unlike human ESCs (hESCs), have a more advanced pluripotent phenotype (“naive”). Accordingly, the induction of such “pause” in cultured mouse and human ESCs has great implications for reproductive and regenerative medicine, but also for expanding our knowledge of certain cellular aspects, such as the dormancy of cancer cells. Cancer cell dormancy is often defined as a reversible transient state of non-proliferating or slow-cycling state, entered by some cancer cells to adapt and survive harsh microenvironmental conditions, eventually implicated in tumor recurrence. Moreover, dormant cancer cells and cancer stem cells are often considered to be similar in the context of radio- and chemoresistance, and also in the sense that the dormant state can be an adaptive cell stress response of cancer stem cells [1,2]. Accordingly, the concept of cancer stem cells underlying the formation of cancer cell dormancy can be akin to diapause mechanisms in ESCs. This is supported by the latest data indicating that blastocysts that are naturally suspended in diapause in vivo and paused blastocysts ex vivo demonstrate a pronounced reduction in mTOR activity and an increase in autophagy activation in a similar manner with dormant cancer cells [3,4,5]. Accordingly, we suggest that a deepening analysis of mTOR pathway regulation in ESCs, especially during diapause, could have important implications on our understanding of the phenomenon of cancer cell dormancy.

**Figure 1:**
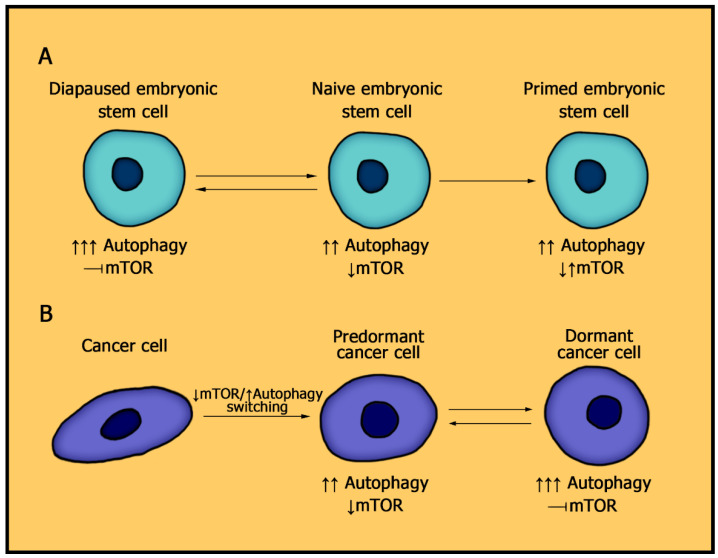
Schematic of review concept. Embryonic stem cells (ESCs) can be exposed to diapause—a prolonged stop of proliferation, followed by its resumption under favorable conditions. Naive ESCs can be isolated from diapaused embryos of mice and, therefore, give rise to all cell types of adult organisms, while primed ESCs obtained from human embryos are more differentiated and have more limited regenerative potential. The low mechanistic target of rapamycin (mTOR) activity in ESCs is correlated with a high autophagy flux, which is more upregulated in naive ESCs. Primed ESCs cannot be exposed to diapause due to the absence of a permissive mTOR signaling profile; in other words, due to inconsistency of signaling pathways converging on the mTOR pathway downregulation. The mechanisms of mTOR suppression are permitted for naive ESCs entry into diapause. Based on the above, we can assume that a similar principle also works in the formation of cancer cell dormancy.

Target of rapamycin (TOR) is a serine–threonine kinase that was originally identified in *Saccharomyces cerevisiae* and then found to be highly conserved among eukaryotes [6]. Mechanistic TOR (mTOR), previously known as Mammalian TOR, together with ATM, ATR, and DNA-PK kinases, belongs to the PIKK family. Although ATM, ATR, and DNA-PK are involved in the cellular DNA damage response, mTOR is implicated in the regulation of cell growth (cell size control) and protein synthesis [7]. Deletion of the mTOR gene results in embryonic lethality shortly after implantation [8,9]. mTOR null blastocysts have an almost normal phenotype but an impaired ability to form trophoblasts, and cells isolated from the inner cell mass (ICM) fail to proliferate when cultured in vitro [8,9]. Hence, the activity of mTOR is essential for embryonic development after the formation of a blastocyst, reflecting the significance of mTOR in cell development. Based on this fact, complete mTOR reduction in differentiated cells is expected to lead to cell death, while under defined conditions, diapause in ESCs will be induced. Therefore, it can be proposed that only cancer stem cells, which are similar to ESCs in the main molecular characteristics, including the expressions of pluripotent factors, can undergo cancer dormancy by a similar mechanism with diapause. The data of RNA-seq revealed that dormant cancer cells, defined by authors as a reversible drug-tolerant persister, share significant similarities of transcriptomes with paused ESCs and in vivo diapaused embryos [4]. In this respect, resistant tumor cells with irreversible cell cycles were transcriptionally distinct from dormant cancer cells [4]. Logically, cancer cells, before committing to a dormant state, are obliged to have the similar to ESCs profile of the mTOR pathway, inhibition of which would allow entering diapause. For this reason, pluripotent cells can be considered as a relevant model for the identification of specific pathways that drive dormant state formation via the mTOR pathway. To that end, it is necessary to clearly understand how the mTOR pathway functions in pluripotent cells, providing entry into diapause. In this review, we analyzed the functional activity of the mTOR pathway in embryonic stem cells in detail, including the antagonistic autophagic pathway that is essential for diapause state formation and prolongation. We did not address the analysis of the mTOR pathway in cancer cells, in particular in dormant cancer cells, as it is a separate theoretical study and beyond the scope of this study. The purpose of this review was to analyze the mTOR pathway in pluripotent cells in order to define the hypothetical mTOR pathway profile underlying cell dormancy in common, with subsequent application of it in the field of cancer research. The conception of the review is presented in Figure 1 and is explained throughout the entire text.

## 2. The mTOR Pathway in ESCs

The mTOR signaling pathway is known to be involved in many cellular functions, such as cell proliferation, metabolism regulation, and cell survival. In addition, it plays a pivotal role in cellular differentiation during embryogenesis. Here, we discuss the components of the mTOR signaling pathway in embryonic stem cells and the main regulators of its function. Furthermore, we refer to the significance of the mTOR pathway in preserving the stemness of pluripotent cells, which might be a major characteristic of cancer stem cells, or dormant cancer cells acquiring a stem-like profile, to enter the dormant state and survive the toxic effects of multiple cancer therapies.

Previous studies established that mTOR knockout is lethal during early murine embryonic development, and the mutant embryos die at around 6.5 to 7.5 post coitum [8,9]. However, upon intraperitoneal injection of the mTOR inhibitor rapamycin into normal pregnant mice from E5.5–E8.5, most regions of these embryos developed normally [10]. Moreover, mESCs exposed to rapamycin are inhibited but do not stop proliferation in comparison to mESCs with conditional mTOR deletion [9]. This is due to the fact that mTOR kinase is the catalytic subunit of at least two distinct signaling complexes, referred to as mTOR complex 1 (mTORC1) and mTOR complex 2 (mTORC2). Rapamycin allosterically inhibits the activity of the mTORC1, while mTORC2 is rapamycin insensitive. Thus, rapamycin treatment of both blastocysts and ESCs incompletely inhibits the overall mTOR activity, therefore demonstrating a weaker effect compared with mTOR deletion in embryos. The complexes appear to participate in different pathways and, thus, have different cellular functions.

mTORC1 complex consists of the common mTOR kinase and specific components—Raptor, mLst8, DEPTOR, and PRAS40—and generally activates anabolic processes, such as protein synthesis, and downregulates catabolic processes, such as autophagy. The conserved function of the mTORC1 pathway is to integrate nutritional, growth factors, and different stress signals with cell proteostasis. Canonically, activated mTORC1 regulates mRNA translation to promote increased protein synthesis, in part, by phosphorylating S6K1 and 4E-BP1 targets. In turn, mTORC2 consists of the common mTOR kinase and Rictor, mLst8, mSin1, DEPTOR, and protein associated with Rictor 1 and 2 (Protor1/2). mTORC2 does not respond to nutrients, but it is sensitive to growth factors and insulin via PI3K-dependent mechanisms [11]. While mTORC1 signaling is well characterized, mTORC2 is relatively poorly understood, especially in ESCs. Due to the lack of sufficient data on the role of the mTORC2 complex in ESCs, we mainly discuss mTORC1 in what follows.

It is considered that the functions of mTORC1 and mTORC2 are closely related but can differently regulate cell fate. This is partly confirmed by the study of the role of these complexes in embryogenesis. Proteins Raptor and Rictor, the positive regulators of mTOR, appear to serve as adaptor proteins that mediate substrate specificity for each complex, and gene knockouts of these proteins are good tools for distinguishing mTORC1/2 functions in embryonic development and ESC propagation. It has been found that Raptor-deficient embryos die shortly after implantation and that explanted Raptor*^−/−^* demonstrate the same proliferation defects as mTOR*^−/−^* embryos [8,9]. Mouse embryos that lack Rictor develop normally until E9.5 and then exhibit growth arrest and die by E10.5–E11.5 [12,13,14]. The exact cause of death in Rictor-null embryos is not obvious but most probably arises due to defective fetal vascular development. In support of this assumption, there are data indicating the involvement of Rictor/mTORC2 in cardiomyocyte differentiation in mESCs in vitro [15,16]. Thus, mTORC1 predominantly functions in early development and mTORC2 is essential for later stages of differentiation until vascular development. These findings also give reason to believe that the formation of pluripotent cells (ICM-derived cells) in embryogenesis can occur in the absence of mTORC1/2, but for further proliferation and differentiation in vivo, the activity of both complexes is required.

**Figure 2:**
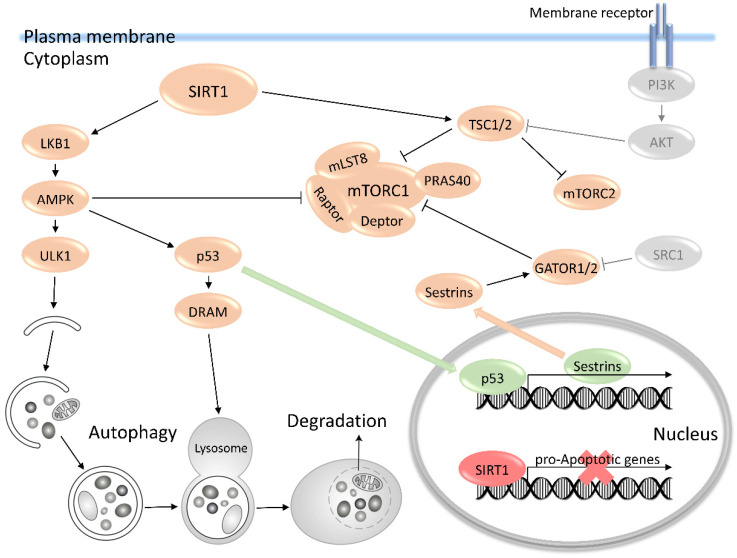
The mTOR pathway profile in ESCs; generalized chart. The PI3K/AKT signaling activates mTOR kinase under the action of different factors: insulin-like growth-factor IGF, fibroblast growth factor FGF, etc. This pathway is downregulated in pluripotent cells. In addition, TSC1/2 is upregulated and suppresses the activity of mTORC1 and mTORC2 complexes. Support of TSC1/2 functional activity also comes from SIRT1 deacetylase, which coordinates autophagy via LKB/AMPK pathway. The main restrained mTOR activity signaling pathway is provided by SIRT1/AMPK/ULK1 pathway. AMPK is activated by SIRT1-dependent acetylation of LKB1. Activated AMPK phosphorylates autophagy-inducing ULK1 complex. Moreover, AMPK has an inhibitory activity on the mTORC1 complex restraining surplus anabolic processes. AMPK also activates p53 that translocates to the nucleus and upregulates *DRAM1* and *Sestrins* gene expression. Proapoptotic p53 activity is suppressed by SIRT1 deacetylation. Lysosomal membrane protein DRAM1 stimulates autophagy, while Sestrins proteins stimulate the activation of the Gator1/2 complex, by which mTORC1 is inhibited.

Interestingly, the requirements for mTOR signaling in hESCs are different from those in mESCs, because inhibition of the mTOR pathway induces differentiation in human pluripotent cells in vitro [17]. A critical distinction between mouse and human ESCs in terms of the signaling requirements for their self-renewal and pluripotency is well established: mESCs isolated from ICM are stable in vitro in the naive state, which resembles the pre-implantation stage of embryogenesis, while hESCs are closer to the primed state, corresponding to the post-implantation stage [18]. Epigenetic, transcriptomic, and metabolic differences between naive and primed ESCs were uncovered in recent studies [19]. Primed hESCs are thought to depend on glycolytic metabolisms and have a low mitochondrial respiratory capacity, while naive mESC have a bivalent metabolism that is characterized by active mitochondria and a high glycolysis level, in addition to their ability to switch between glycolysis and OXPHOS in response to changes in environmental conditions [20]. The concept of identifying the mTOR pathway as the robust controller of the cellular metabolic program and defining cell-tissue specificity, including cancer cells, was recently proposed [21,22]. mTORC1 signaling underlies a coordinated metabolic network in cells and mediates the emergence of respective adaptive mechanisms under different stress conditions, by accordingly linking cellular metabolism to cell physiology, such as growth, survival, division, migration, and differentiation. These findings provide evidence that the mTOR signaling profile underlying cell metabolism is crucial for stem cell proliferation and differentiation but is different in naive mouse ESCs and prime human ESCs. In accordance with this, treatment of epiblast-like pluripotent stem cells with the mTOR inhibitor INK128 does not induce diapause, while it effectively mimics hormonally induced diapause in naive cells [5]. Therefore, for long-term propagation of naive and primed ESCs, both mTORC1 and mTORC2 activity are required, but in addition, naive ESC state demands a stringent balance of the mTOR activity for diapause entry (Figure 2). Accordingly, more studies should be conducted on mESCs in the diapause state to indicate the major regulators of this phenomenon and highlight the exact dynamic mechanism underlying the entry and exit from diapause, which consequently, may carry significant reflections to understand the cancer cell dormancy.

## 3. The mTOR Pathway versus Autophagy in Pluripotent Cells in Detail

As previously mentioned, one of the most important functions of mTOR is to regulate metabolism and cellular energy, which are mainly regulated by controlling mRNA transcription and protein synthesis, as well as by regulating autophagy. Autophagy is a cytoprotective response to stressful circumstances such as nutrient deprivation and impaired cellular homeostasis, providing the cell with essential amino acids and energy. Accordingly, we discuss the regulation of autophagy in embryonic development, which is critical to maintaining cellular homeostasis in ESCs during proliferation and differentiation, and especially, during unfavorable conditions such as diapause. We also highlight the role of the mTOR pathway in regulating autophagy, in addition to other main participating regulators in this context. 

The autophagy is massively induced in the oocyte in a short time after fertilization, which undergoes a large-scale intracellular rebuilding, the so-called process of maternal-to-zygotic transition [23,24]. Given that the time of early embryonic development is quite fast, and the degradation of proteins using the ubiquitin/proteasome system is ineffective, it is reasonable to assume that autophagy plays a key role at this stage of embryonic development. The fact is that mouse blastocysts show increased autophagy when delayed implantation is induced by estrogen removal [25]. The prolonged dormancy of blastocysts accompanies an extended period of autophagic activation, which is the main mechanism of blastocyst survival under unfavorable conditions. This suggestion is confirmed by an increase in apoptosis in dormant blastocysts after 3-MA treatment (a widely used inhibitor of autophagy), suggesting that cell death and other cellular destructive processes occur in case of blockade of autophagy [25,26]. Therefore, ESCs derived from ICM under diapause demonstrate a high autophagic activity correlated with their pluripotency.

mTOR is known to suppress autophagy, and therefore, it can be assumed that blastocysts and isolated ESCs possess a higher autophagic flux due to decreased activity of the mTOR pathway or increased expression of core autophagic components. A few recent studies have confirmed that pluripotent cells are characterized by reduced levels of global translation compared with differentiated cells, due to downregulation of mTOR pathway activity [27,28]. In addition, ESCs demonstrate a higher basal autophagic activity in comparison with terminally differentiated cells, like neurons or fibroblasts [29,30]. Thus, the metabolic balance of ESCs is shifted to catabolic processes, and disturbances in this balance, for example, by the expression of constitutively active p70S6K, will induce differentiation in pluripotent cells [27]. The indicated high autophagic flux maintains the identity of ESCs by guarding their self-renewal and pluripotency capacity. SIRT1/AMPK/ULK1 signaling appears to be a key mechanism of increased autophagy level in ESCs (Figure 2).

Recent studies have shown that Sirtuin 1 (also known as SIRT1) plays a pivotal role in the self-renewal and differentiation of various stem cells [31,32,33,34]. SIRT1 is an evolutionarily conserved NAD^+^-dependent deacetylase that plays an essential role in the regulation of different cellular functions. The activity of the mTOR pathway in SIRT1-deficient mouse embryonic fibroblasts increases [35]. Thus, one of the aspects of mTOR regulation in pluripotency and differentiation could be via deacetylase SIRT1, which is abundantly expressed in ESCs [35]. A model of negative regulation of mTOR signaling by SIRT1 was proposed, mediated through its association with TSC2 [36]. The protein complex TSC1/2 has been reported to have an inhibitory function on mTORC1. It has been recently established that *Tsc2 ^−/−^* ESCs from Eker rats possess characteristic features of ESCs, including expression of pluripotency markers, long-term self-renewal, and the capacity to differentiate into derivatives of all three germ layers [37]. TSC1/2 deficiency resulted in preserved mESCs homogeneity and in strong resistance to differentiation upon differentiation stimulus, probably indicating the suppression of the mTOR activity and subsequently differentiation-promoting programs [38]. On the contrary, under *Tsc1/2* deletion, the mTORC1 complex is active, and it could be assumed that inhibition of differentiation can occur via the loss of the mTORC2 activity. In support of this suggestion, there are data indicating that mTORC2 kinase activity is impaired in cells lacking a functional TSC1/2 complex [39]. Therefore, the TSC1/2-mediated suppression of mTORC1 is not a key mechanism for maintaining ESC identity, but it is essential for cell development.

The main regulation of mTOR via SIRT1 also involves other mechanisms, in particular, the activation of AMPK kinase. SIRT1 may act upstream of AMPK, and it can potentially deacetylate and activate the major AMPK activating kinase LKB1 [40]. AMPK inhibits mTORC1 through direct phosphorylation of Raptor, however, one of the most important AMPK-dependent events is direct phosphorylation and activation of ULK1 [41,42]. In mammals, phosphorylation of ULK1 by AMPK is required for ULK1 function in the response to nutrient deprivation. The ULK1 complex initiates autophagy by promoting the formation of double-membrane structures known as the phagophores. Both ATG13 and FIP200 are required for stimulation of ULK1 kinase activity and are critical for correct ULK1 localization on the membrane [43]. In turn, the ULK1 complex is negatively regulated by mTOR, which directly interacts with ULK1 and phosphorylates it and ATG13 [44]. ULK1 is highly expressed in ESCs, and its kinase activity is critical for pluripotency regulation [30]. The significant enhancement of the expression of genes involved in the ULK1 autophagy initiation complex (ULK1, ATG13, FIP200, and ATG101) was demonstrated by comparing the expression of core molecular machinery genes between iPSCs and somatic fibroblasts [45]. ULK1 deficiency dramatically decreases the autophagic flux in mESCs and inhibits the self-renewal and pluripotency of mESCs. The ULK1 activity is mainly maintained by AMPK-dependent phosphorylation on Ser555 and Ser317. In addition, ULK1 phosphorylation on Ser555 by AMPK, but not on Ser757 by mTOR, was detected to be significantly higher in pluripotent stem cells than in somatic MEFs [30]. The authors of this study suggested that constitutive activation of ULK1 by AMPK is an intrinsic signaling pathway in ESCs to regulate their identity under normal physiological conditions. This hypothesis was confirmed by the results obtained from the stable mESC line with doxycycline-inducible *Ulk1* gene expression [46]. *Ulk1* overexpression enhances the AMPK/ULK1 signaling pathway activation that is accompanied by an increased pluripotency network in mESCs. Therefore, SIRT1/AMPK/ULK1 signaling underlies autophagy in pluripotent cells and predominates the mTOR pathway in pluripotent cells. 

AMPK can also suppress mTOR through p53 activation, which was demonstrated in resveratrol-treated mESCs [47]. This occurs through AMPK-dependent p53 phosphorylation; subsequently, p53 translocates into the nuclei and transactivates *Sestrin1* and *Sestrin2* genes, which are mTOR inhibitors proteins, and additionally stimulates autophagy in a DRAM-dependent manner. Currently, Sestrins proteins are defined as novel molecular links that restrict mTORC1 pathway activation in response to stress stimuli and suppress the mTORC1 via complex interaction with the GATOR1/2 [48,49,50]. It was shown that genetic deletion of the GATOR1 complex increases the heterogeneity of mESCs and accelerates differentiation under conditions of the differentiation [38]. Gator1*^−/−^* ESCs showed the opposite phenotype compared with Tsc2*^−/−^* cells, suggesting the significance of the GATOR1/2-dependent inhibition of mTORC1 in the regulation of ESC identity. This finding is also in agreement with the following data: It was identified that Src is a critical activator of mTORC1 and acts upstream of Gator1; accordingly, mTORC1 activity is higher in cells having endogenous activation of Src [51]. Src-family kinase signaling is required for the initiation of the differentiation program in mouse and human ESCs, and ESCs, owing to non-functional Src signaling, restrain the mTOR pathway due to Gator1 activation [52,53].

Based on the above, naive and primed ESCs demonstrate pronounced dependence on autophagy, compared with the mTOR pathway. Nevertheless, ESCs must demonstrate the downregulated mTOR signaling, permissive for diapause entering. However, the mTOR pathway profile in primed ESCs appears to be different from the one in naive ESCs, as the last can be exposed to diapause during inhibition of mTOR [5]. This may explain why not every mTOR inhibition could induce diapause in mESCs or entry of cancer cells into dormancy, as a defined profile of mTOR regulation should be met in advance.

Previous observations showed the high dependence of ESCs on autophagy machinery to maintain its integrity and identity in various conditions, including diapause. SIRT1, AMPK, and GATOR1/2 have also been shown to have major roles in regulating mTOR and autophagy activities in ESCs. However, literature data are deficient about the role of these proteins in promoting or inhibiting diapause in ESCs. Accordingly, further studies should be conducted using genetic modifications tools to analyze the involvement of these proteins in promoting or suspending diapause, which may lead to promising results that could be applied to the dormant state of cancer cells. It is also suggested to conduct such experiments on cancer cells and analyze their ability to enter or exit a dormant state, in addition to observing whether it is related to acquiring a stem-like profile. 

## 4. The Key Upstream Regulation of the mTOR Pathway and Autophagy in ESCs

The mTOR signaling pathway is integrated and undergoes crosstalk with multiple proteins of different intracellular pathways in order to coordinate various fundamental processes that highly depend on its level of activation. Of these proteins, FLCN, TFEB, and FOXOs are also considered to modulate mTOR activity, but also the activation of autophagy, and interestingly, this has a major impact on the pluripotent status of stem cells and also diapause. Therefore, a detailed analysis of their function is covered below.

Recently, genome-wide CRISPR-KO screening identified that one of the main differences in the mTOR pathway regulation between naive and primed ESCs involves FLCN protein [38,54]. FLCN (folliculin; BHD gene) is a protein without well-defined functions in cells but with pronounced antitumor function in kidneys. It has been revealed by experiments on FLCN-deficient renal cells that they are tumorigenic, also shown in mice with BHD heterozygous knockout, which develop kidney cysts and renal solid tumors as they aged [55,56,57]. Deficiency of tumor suppressor FLCN leads to the activation of the mTOR signaling pathway in cells; nevertheless, Flcn*^−/−^* mice are embryonic lethal soon after implantation similar to mTOR-depleted mice. This confirms the significance of the FLCN–mTOR signaling axis in early embryogenesis and cell development [55,58].

FLCN is a highly conserved GTPase-activating protein that forms a complex with FLCN interacting proteins 1 and 2 (FNIP1/2). Classically, mTORC1 is transferred to lysosomes and localized there via Rag GTPases and can be activated by Rheb GTPases. Intriguing localization of mTORC1 at lysosomes is currently widely discussed and appears to prevent anabolic processes. Accordingly, it is necessary to prevent mTOR localization on lysosomes in order to limit its activity, for example, through Sestrins and GATOR1/2 complex [48]. Lysosomal biogenesis is regulated by transcription factors TFEB and TFE3 that promote expressions of many lysosomal genes and critical regulators of autophagy. FLCN activation leads to translocation of TFEB and TFE3 from the nucleus to the cytoplasm on the lysosomes, where mTORC1 phosphorylates and inactivates these factors, abrogating TFEB/TFE3-mediated transcriptional activation of autophagy and fusion of lysosomes with autophagosomes.

TFE3 is localized in the nucleus of naive ESCs and in the cytoplasm in primed ESCs, demonstrating a significant difference in the regulation of the mTOR pathway in these cells [59,60]. Accordingly, mTORC1 does not downregulate autophagy via controlling the activity of TFEB through its cytoplasmic retention in naive cells. It can be assumed that the FLCN pathway is not functional in naive cells, and the mTOR pathway is not upregulated by FLCN. This proposition is evidenced by CRISPR KO screening obtained that FLCN is only critical for the exit from the human naive pluripotent state [60]. FLCN KO hESCs maintain the naive pluripotent state but cannot exit it, since the critical transcription factor TFE3 remains active in the nucleus and the TFE3 transcriptional program is upregulated in cells. The localization of TFE3 in the nucleus in ESCs may be one of the main intracellular programs to restrict mTOR activity and provide high autophagy flux in naive pluripotent cells. Interestingly, the exit of TFE3 from the nucleus in ESCs is controlled by mTORC1, while mTORC2 activity is inessential for this, which emphasizes the mystery of mTORC2 [60]. Functional analysis revealed that mediating the cytoplasmic localization of TFE3 by FLCN in primed ESCs through *FLCN* gene expression is similar in naive and primed ESCs, suggesting the functional inhibition of FLCN protein in naive ESCs [54,59].

**Figure 3:**
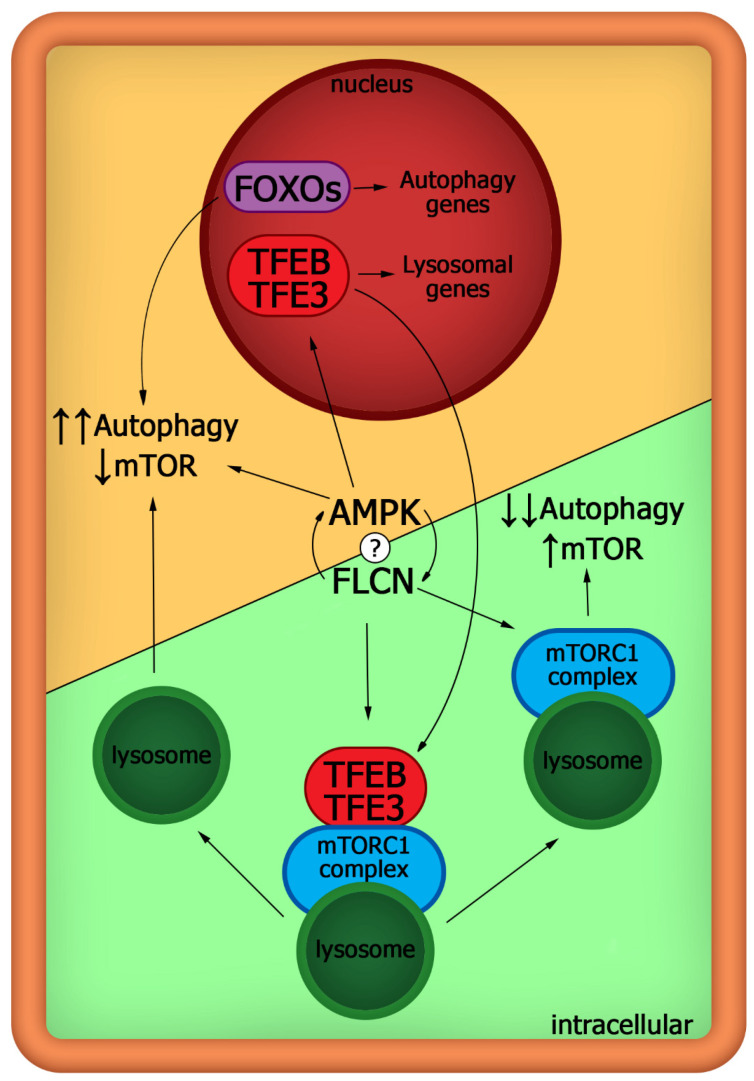
Schematic representation of the key regulation of the mTOR pathway in ESCs during the transition from the naive state to the primed state and vice versa. A high level of autophagy in naive ESCs is maintained by two linked transcriptional modules FOXOs and TFEB/TFE3, which are under the control of AMPK kinase. The mTOR pathway is downregulated and does not interfere with TFEB/TFE3 nuclear localization. Activated FLCN pathway disintegrates AMPK-dependent regulation of autophagy and, as a GTPase activating protein, it recruits mTORC1 and TFEB/TFE3 to lysosomes where mTORC1 inhibits the latter. mTORC1 is activated on lysosomes by Rag and Rheb GTPases and triggers anabolic processes.

Currently, it is not clear which mechanism is involved in the FLCN/mTOR pathway restriction, but hypothetically, it may be via AMPK kinase that is highly upregulated in ESCs (Figure 3). AMPK may promote dephosphorylation and nuclear translocation of TFEB and TFE3, protecting them from the inhibitory effect of mTOR in cells [61,62]. In addition, AMPK directly phosphorylates FOXO transcription factors at six regulatory sites, which provides additional upregulation of autophagy in cells [63].

One of the main factors controlling the high autophagy activity in ESCs exists at the level of FOXO transcription factors. It was identified that FOXO1 drives an autophagy machinery gene program to maintain high autophagic flux in ESCs [45]. At the same time, FOXO1 directly targets both pluripotent genes and autophagic genes, coordinating the autophagy gene program with the pluripotency network in ESCs. FOXO3 was also identified to regulate autophagy through directly upregulating the expression of autophagic genes in stem cells, and furthermore, it was shown that FOXO3 also mediates FOXO1-dependent autophagy [64]. FOXO3A was determined as critical to maintaining a gene expression program that adjusts hematopoietic stem cells for rapid induction of autophagy upon starvation, which is necessary to mitigate the energy crisis and ensure their survival [65]. Approximately, the same mechanism of a protective autophagic gene expression program via FOXO3 was discovered in dormant ESCs emerged during neural differentiation [66]. In this report, it was shown that a subpopulation of mESCs transits into a dormant state during neural differentiation in a FOXO3-dependent manner without compromising pluripotency. In addition, previous studies have reported that FOXO3 suppresses MYC-dependent transcription and reduces the expression of MYC [67,68]. *Myc*-depleted ESCs enter a state of dormancy similar to embryonic diapause, and c-Myc regulates their entry and exit from the dormant state [69]. Moreover, hypoxia induces the dormant state in oocytes through FOXO3 and, correspondingly, the dormant state of these cells in vitro can be triggered by overexpression of constitutively active FOXO3 [70]. Interestingly, the state of dormancy detected in several types of cancer stem cells is also maintained by FOXO3 [71]. Therefore, FOXO3-mediated regulation of cell dormancy is likely a common event among normal stem cells and cancer stem cells as an evolutionary adaptive strategy of cell quiescence.

Hence, the catabolic response regulated by the AMPK-controlled module is prevailed in ESCs and is essential for diapause induction and prolongation. This module can be disturbed by FLCN protein activation because AMPK is inhibited by FLCN-binding partners FNIP1 and FNIP2 proteins. As FLCN/FNIP1,2 axes are evolutionarily conserved negative regulators of AMPK, the loss of FLCN leads to constitutive activation of AMPK [72]. Therefore, AMPK/FOXOs and FLCN/mTOR pathways can be key signaling modules contributing to the equilibrium of ESCs homeostasis by coordinating the mTOR pathway and autophagy. Currently, these signaling pathways are increasingly being discussed in the context of understanding the biology of the development of certain tumors, and it is supposed to be investigated more carefully [73]. Accordingly, these studies should involve the development of relevant models of dormant cancer cells, to analyze the coordination between these regulators during dormancy, the dependence and relationship between their level of activation with the status of dormancy, as well as the response of cancer cells to various therapies.

## 5. The Role of mTOR Signaling Pathway and Autophagy in Dormant Cancer Cells

Cancer recurrence is significantly driven by the ability of some cancer cells to survive and confront the cytotoxic effects of various therapeutic approaches, which primarily target highly proliferating cancer cells. Several studies have reported that these residual cancer cells are often slowly cycling or non-proliferating cells, in other words, quiescent or dormant cells. While conventional therapies mainly target nuclear DNA in rapidly dividing cancer cells, causing high DNA damage and cell death, the dormant state of residual cancer cells abolish the most effectiveness of therapeutic agents. Interestingly, resistant cancer cell populations were shown to be able to exit the state of dormancy and return to proliferate intensively following treatments withdrawing, eventually leading to tumor reformation and the onset of recurrence.

To date, the origin and characteristics of dormant cancer cells are not fully understood. Multiple studies identified the existence of these cells in both solid tumors, including breast [74], glioblastoma [75,76], pancreatic [77], lung [78], colorectal [79,80], ovarian cancers [81], and hematologic malignancies, such as leukemia [82] and melanoma [83]. Dormant cancer cells are thought to drive the non-genetic therapy resistance, and they generally comprise a small subset of the primary tumor (0.3–5%) [84]. In turn, cancer stem cells are also insensitive to traditional therapies by virtue of their slow-proliferating rate. In addition, their potential to escape from the immune response and fuel tumor relapse is also known [85]. Accordingly, cancer stem cells demonstrate significant similarities with dormant cancer cells, in terms of rareness, slow cycling, and capacity to recover and reinitiate tumors [86,87]. Thus, further investigation into the molecular mechanisms underlying dormancy and therapy resistance could introduce promising therapeutic strategies to target dormant cancer cells and minimize the risk of resistance and tumor relapse.

The engagement of multiple factors in regulating cancer cell dormancy has been highlighted. Of these factors, PI3K/AKT/mTOR signaling pathway is increasingly believed to play a major role, as it is the most commonly activated signaling pathway in human malignancies, implicated in both tumor progression and resistance. For instance, the upregulation of the PI3K/AKT/mTOR pathway was observed in 70% of ovarian cancers [88] and 30–40% of breast cancers [89]. Moreover, PI3K/mTOR pathway is indicated as one of the main causes of prostate cancer resistance to therapy [90]. It also mediates resistance in other solid and hematologic malignancies, such as breast [91], colorectal [92], lung [93], and melanoma cancers [94]. The interest in targeting the PI3K/mTOR pathway as a therapeutic strategy is remarkably increasing, in particular after the recent finding that the dormant state entered by some cancer cells as a result of stress-inducing factors is very similar to diapause, the mechanism used by ESCs to survive harsh microenvironmental conditions [3,4]. These recent studies have demonstrated that dormant cancer cells, in patient-derived breast and colorectal cancer models, exhibit a very similar transcriptional profile of the embryonic diapause [4,95]. Rehman et al. showed that colorectal cancer cells treated with Irinotecan (CPT-11) employed the same survival strategy of mESCs to escape from cytotoxic effects of therapy. This diapause-like dormant profile was also mainly characterized by mTOR reduction and autophagy activation [4]. Interestingly, dormant cells returned to proliferate when the treatment was terminated, illustrating the reversibility of this state, as in embryonic diapause. Furthermore, the use of only mTOR inhibitor INK128 was sufficient to induce the entry of some cancer cells into the diapause-like dormant state, while withdrawing the treatment resulted in cell growth resumption. These findings suggest that mTOR targeted inhibition, similar to applying CPT-11, causes colorectal cancer cells to enter the reversible diapause-like state [4]. Similar results were obtained by Kim et al. in a study showing that TANK-binding kinase (TBK1), by interacting with mTOR and inhibiting its function, stimulates prostate cancer cell dormancy in the bone marrow niche. Interestingly, the rapamycin-mediated inhibition of mTOR also resulted in an enlarged population of dormant cancer cells and, furthermore, increased their resistance to chemotherapy [96]. This was supported by another study demonstrating that the reduction in PI3K/AKT/mTOR signaling was correlated with triggering the dormant state in disseminated cancer cells in the bone marrow, in addition to their expression of dormancy markers, such as the low expression of cell proliferation markers ki67 and PCNA [97]. Indeed, other studies also indicated the involvement of the PI3K/AKT/mTOR signaling pathway in mediating the dormant state in multiple cancer models. For instance, inhibiting the pathway activity by a specific AKT inhibitor, Akti-1/2, induces the transition of ovarian cancer cells into a dormant state [98]. Additionally, the long-term exposure of breast cancer cells to hypoxia resulted in the downregulation of mTOR signaling, which was associated with an increased number of dormant populations [99]. Similarly, treating gastrointestinal stromal tumor cells with Imatinib resulted in a reduction in mTOR signaling and stimulation of entry into dormancy [100]. In an ovarian cancer model, the reexpression of DIRAS3 (also known as ARHI, a tumor suppressor that is commonly downregulated in breast and ovarian malignancies [101,102]), although inducing significant cell death, was shown to inhibit mTOR and its downstream effector p70S6K, plus activating autophagy, which cause a subpopulation of cells to enter a dormant state [102]. On the other hand, stimulation of PI3K/AKT/mTOR signaling corresponded with fewer dormant cancer cells in tumor burden and improved response to cancer therapy [96]. Previous observations highlight the significant role of mTOR signaling in mediating therapy resistance of cancer cells via entering a diapause-like dormant state depending on mTOR inhibition and autophagy activation, the same way as diapaused mESCs adapt and survive unfavorable conditions. 

In line with mTOR inhibition, ULK1 is dephosphorylated on Ser757, and autophagy is consequently upregulated, stabilizing the diapause of mESCs, as well as the dormancy of cancer cells. Bulut-Karslioglu et al. showed that embryonic blastocysts entered to diapause have a high level of autophagy activation, and the inhibition of ULK1 with SBI-0206965 provoked blastocyst exit from diapause [3]. Similarly, Rehman et al. revealed the dependence of cancer cells entered into diapause-like drug-tolerant persister state on autophagy activation. As previously mentioned, high transcription levels of autophagy-related genes were demonstrated in patient-derived xenografts colorectal cancer cells, which existed in the diapause-like dormant state. This was confirmed by the high expression of autophagy proteins in dormant cancer cells cultured in Vitro [4]. Furthermore, Autophagy inhibition by ULK1 inhibitor (SBI-0206965) or chloroquine, in combination with Irinotecan, resulted in substantial activation of apoptosis and massive cell death compared with each drug separately. In addition, the combinatorial treatment abolished the potential of dormant cancer cells to recover and reproliferate upon treatment cessation [4]. These observations indicate a great dependence of dormant cancer cells on autophagy activation to survive the harsh surrounding conditions. Another key activator of autophagy in cancer cells is the energy sensor kinase AMPK, which phosphorylates ULK1 on Ser555, similar to ESCs. Additionally, as previously discussed, AMPK also downregulates mTOR in low-energy cellular states, by directly phosphorylating mTORC1 and tuberous sclerosis complex 2 (TSC2) that, in turn, phosphorylates mTORC1 and inhibits its activity, resulting in a further induction of the autophagic flux. Indeed, low levels of intracellular ATP primarily exist in dormant cancer cells, leading to the upregulation of the LKB1-AMPK pathway, which was shown to be required to stabilize the dormant state in ovarian and breast cancer models [103,104]. Interestingly, treating proliferating ovarian cancer cells with AMPK activators (AICAR or A-769662) resulted in arresting the cell cycle and entering a quiescent state [103]. In addition, a dormant population of breast cancer was enriched upon chronic hypoxia, where AMPK and autophagy upregulations were essential to maintain the energy balance of dormant cells [99]. These observations further indicate the role of autophagy as survival machinery in different dormancy inducing-circumstances. Transcription factors, such as FOXO3 and TFEB, have also been shown to take their part in autophagy regulation in dormant cancer cells, as in ESCs. In DIRAS3-mediated dormancy of ovarian cancer cells, mTOR inhibition by DIRAS3-induced translocation of FOXO3 and TFEB to the nucleus, which stimulated the transcription of multiple autophagy-related genes [105]. Based on the above observations, the disruption of cancer cell dormancy and embryonic diapause could be achieved through autophagy inhibition, which was supported by numerous recent studies. As previously mentioned, the combination of autophagy inhibitors, SBI-0206965 or Chloroquine, with Irinotecan, resulted in a high level of apoptosis in the dormant population and interrupt their ability to fuel tumor regrowth [4]. Similarly, noticeable cell death of dormant breast cancer cells was observed following their treatment with autophagy inhibitors 3-methyl adenine, chloroquine, or bafilomycin [106]. Another supporting result of the efficacy of inhibiting autophagy during dormancy was obtained by using chloroquine to inhibit autophagy in the SKOv3 ovarian cancer cell line dormant population, resulting in reducing tumor outgrowth [107]. These findings further illustrate the role of autophagy in sustaining the dormancy of cancer cells and indicate the significance of investigating the involvement of the mTOR pathway in driving cancer cell dormancy. Herein, mESCs can serve as a good tool to comprehensively investigate the diapause mechanism, understanding of which may have great implications on understanding the phenomenon of cancer dormancy, and thus, new promising targets and therapeutic strategies may be developed to control the dormancy and reduce the incidence of tumor relapse. 

## 6. Conclusions and Future Perspectives

Until now, there is no complete understanding of the so-called dormant state of cancer cells, which determines the phenomenon of relapse in anticancer therapy. The main problem in defining cancer cell dormancy is that it does not appear to be a mutation-related mechanism, and all cancer cells have the potential to enter into dormancy [4]. Secondly, cancer cell dormancy is often intended as any state of non-proliferating cells that retain their capability to reenter the cell cycle. If we proceed from this definition of dormancy, then the phenomenon of dormancy is also inherent in somatic stem cells, progenitor cells, fibroblasts, and other cells capable of entering a state of quiescence [108,109,110,111]. Moreover, stem cells can be in two diverse dormant states—short-term and long-term, which determines their regenerative potential to varying degrees [112,113]. There is confusion in definitions such as dormant cancer cells, quiescent cancer cells, tolerant cancer cells, persister cancer cells, and resistant cancer cells, which leads to the generalization term “dormant cancer cells”. This problem has already been reported in publications [114,115]. Based on the analysis of the literature about the quiescent state of stem cells and other cells, dormancy is probably of varying degrees, determined by the balance of intracellular mechanisms, including the cell cycle, mitogenic signaling, epigenetic modification, and metabolism [116]. However, strict terminology in the definition of different states of cellular dormancy, and, moreover, the definition of the fundamental cause of cancer cell dormancy, may not be determined due to lack of sufficiently structured data about cellular mechanisms underlying cell dormancy and, even more so, the regulation of different graded depth detected in various cell types. The discovery of the phenomenon of diapause, a natural transient developmental arrest of blastocysts, allows researchers a fresh perspective on cell quiescence (related to normal cells) and cancer cell dormancy.

Loss of mTOR activity is always conjugated with inhibition of cell proliferation, growth, and differentiation. Consequently, depending on the degree of mTOR kinase suppression, cells can slow down or completely stop proliferation and immerse into a dormancy of varying degrees of depth. All these outcomes are caused by different signaling pathways converging in the mTOR pathway downregulation. As we demonstrated in this review, ESCs initially have a low level of mTOR activity, and the inhibition of mTOR removes its residual activity and allows cells to enter diapause—a deep state of proliferative dormancy. Moreover, in ESCs, the metabolic balance is strongly shifted toward autophagy, which is regulated by at least two extensive transcriptional programs FOXOs and TFEB/TFE3 proteins, allowing cells to survive at the expense of internal reserves during diapause. Apparently, cancer cells can use a similar strategy of transition, firstly, into a pre-dormant state, when intrinsic signaling cascades are coordinated on the mTOR kinase similarly to ESCs, and then transfer into a dormant state.

## Data Availability

Data sharing is not applicable.
